# Psychosomatic Symptoms Among Young Carers: A Population‐Based Survey in Finland

**DOI:** 10.1111/scs.70112

**Published:** 2025-09-19

**Authors:** Ella Eronen‐Levonen, Miko Pasanen, Kaisa Mishina, Agnes Leu, Riitta Suhonen, Katja Joronen

**Affiliations:** ^1^ Department of Nursing Science University of Turku Turku Finland; ^2^ Diacony University of Applied Sciences Helsinki Finland; ^3^ Research Centre for Child Psychiatry University of Turku Turku Finland; ^4^ INVEST Research Flagship University of Turku Turku Finland; ^5^ Institute for Biomedical Ethics, Medical Faculty University of Basel Basel Switzerland; ^6^ Turku University Hospital Turku Finland

## Abstract

**Background:**

Young carers provide significant care for their significant others, and this group of people is often hidden in schools and society. Previous research has shown that young carers face several wellbeing deficits. There is little nationally representative research on the health of this vulnerable group of young people.

**Aims:**

This study first examined how young carer background is associated with psychosomatic symptoms among 16–18‐year‐olds in a general population. Secondly, the study analysed how young carer background and socio‐demographic variables explain psychosomatic symptoms when confounding factors are controlled.

**Methods:**

The data source was the School Health Promotion (SHP) study, a nationwide self‐report survey of students (*n* = 61,448) in upper secondary and vocational schools in Finland. Chi‐squared tests and logistic regression analyses were carried out.

**Results:**

Almost 9% of the students had experiences of caring relationships at least monthly, and 5% at least weekly. Psychosomatic symptoms were more frequent among 16–18‐year‐old students who had caring responsibilities at least weekly than among those who had caring responsibilities monthly or less often. In addition, several socio‐demographic variables such as female gender, economic situation of the family, living with only one or without parents, and confounding factors, that is, self‐perceived health and anxiety, were associated with psychosomatic symptoms. After controlling for all factors, this study found that a caring relationship was still associated with a higher frequency of perceived psychosomatic symptoms.

**Conclusions:**

These findings could promote professionals in health promotion, health care, and education, to be more aware of the existence and support needs of young people with caring responsibilities.

## 1 Introduction

International studies and reports reveal that in Western countries, 6% to 16% [1, 2, 3, 4, 5] of children and adolescents under the age of 18 have regular caring responsibilities for their loved ones. These responsibilities often rise due to a family member's physical or mental illness, substance abuse, or other health challenge. A young carer is a young person who provides regular and substantial care to their significant ones and has been called a young carer or young adult carer [6]. This article uses the terms “young carer”, “young person in a caring relationship”, or “one with caring responsibilities”.

The first studies of young carers were conducted in the UK in the late 1980s (e.g., [7, 8]). During the last decades, this invisible and vulnerable group of young people has gradually been revealed across countries everywhere. The phenomenon is still just now being recognised in many countries, and further information is necessary to realise the existence of this group of young people nationally [3]. Young carers have more depressive symptoms [9] and they experience lower levels of well‐being and higher levels of perceived stress than their peers [4]. Caring relationships where the carer is young have been associated with educational difficulties. Also, the quality of young carers' social conditions, health and well‐being, and the number of employment opportunities are lower [10]. We clearly need to understand the concept, experiences, and specific needs of young carers [5, 11].

Caring relationships can lead to increased stress [12], which may be associated with higher levels of psychosomatic symptoms [13]. Psychosomatic symptoms vary, and a wide variation exists in the definitions of somatic and psychosomatic symptoms. Ordinary psychosomatic symptoms include headache, neck and back pain, stomach pain, rash and repeated infections, and symptoms of depression, irritability, anger, nervousness, and sleeping disorders [14]. If the diseases cannot be diagnosed, psychosomatic symptoms caused by environmental factors, such as one's life situation, should be considered [14]. Tossani et al. [13] reported that this special issue on caring represents a much‐needed advancement, especially from a psychosomatic perspective. There is still limited understanding of the psychosomatic and longitudinal mechanisms through which both physical and mental health problems of significant others impact the next generation [13]. However, a lack of research exists, especially concerning the psychosomatic symptoms of young carers. Young carers are at a higher risk of developing mental health conditions due to the constant stress and emotional strain of caregiving than their peers without caregiving responsibilities [15]. Addressing psychosomatic symptoms can help identify and manage mental health concerns in this vulnerable population of young carers.

In Finland, there is a lack of research concerning young carers. Recognising the phenomenon of young carers and the possible burdens that may arise from it in a Finnish perspective may also inform international research and facilitate international cooperation to improve the situation of young carers all over. It is already known that young carers have more challenges than young persons without a caring relationship. At an individual level, gaining all possible support in a caring relationship is essential. On a large scale and at a professional and societal level, providing knowledge about the psychosocial well‐being and ill‐being of young carers is critical to developing support and services for young carers and informing further policy recommendations. According to Matzka and Nagl‐Cupal [16] it is important for health and social care providers to actively recognise the often‐concealed role children and adolescents may play in caring for relatives and to form supportive and trusting relationships with them [16]. First, young carers and their reactions, such as psychosomatic symptoms, must be identified in, for example, school health care, and second, this phenomenon must be seen in society. This study examines the prevalence of psychosomatic symptoms and the associated factors in the lives of young caregivers.

## 2 Purpose of Study

This study aims to examine how the young persons' caring relationship is associated with psychosomatic symptoms among 16–18‐year‐olds in a general population. Another aim is to analyse how socio‐demographic variables and young carer status explain psychosomatic symptoms when confounding factors are controlled. The study focuses on persons aged 16–18 due to their critical life transitional phase to adulthood. The present study contributes to the research base by studying the associations between care relationships and psychosomatic symptoms in a nationally representative sample and from the under‐researched subjective health perspective.

## 3 Methods

### 3.1 Data Source, Sample, Procedures, and Measurements

The data source is the School Health Promotion (SHP) study from 2019, and it was used with permission of the Finnish Social Science Data Archive. SHP is a nationwide survey conducted by Finnish Institute for Health and Welfare every other year, from March through April. This specific study's target group is part of the survey and consists of first‐ and second‐grade students from vocational and upper secondary schools in Finland in 2019 [17]. Associations between a caring relationship and psychosomatic symptoms were studied using this data. In 2019, data from the SHP study was assessed to cover approximately 70% of upper secondary students in Finland. The coverage of vocational school students was not documented during this period. It is assumed that the participants represent the general population of secondary high school and vocational school students. Of this group, almost 9% had experiences of caring relationships at least monthly and 5% at least weekly.

### 3.2 Measures

#### 3.2.1 Psychosomatic Symptoms

Psychosomatic symptoms were measured through the Adolescent Health and Lifestyle Survey (AHLS) questionnaire, which includes a scale for psychosomatic symptoms. The question was, “Have you had some of the following symptoms during the past half year?” The specific symptoms included neck or shoulder pain, lower back pain, stomachache, sleeping difficulties, headache, or tiredness. The respondents rated the items using a 4‐point Likert scale: *Seldom or never, Once a month, Once a week, or Almost daily*. The scale is part of a larger questionnaire, which the Adolescent Health and Lifestyle Survey has used since 1977 in Finland (https://ghdx.healthdata.org/series/finland‐adolescent‐health‐and‐lifestyle‐survey).

#### 3.2.2 Caring Relationship

Students were asked to answer the following structured question to measure their *Caring relationship*: “Are you helping or caring for a family member or some other person close to you who has, for example, a serious illness or injury or who is very old?” The response options were: *This situation or need for help does not concern my family, A few times during the year, Every month, Every week*, and *Daily or almost daily*. This study reclassified the question into three categories: *None, A few times during the year/monthly, Weekly or Daily*. We think caring for a loved one at least once a week signifies a regular commitment to providing care, even if the time spent may vary. The predictability of this commitment makes it a regular activity. This question was used for the first time in the School Health Promotion (SHP) study in 2019, and it was developed by an international panel of experts and researchers in the field of young carers. The group was set up by the Finnish Institute of Health and Welfare. M. Heino (personal communication 15.4.2023).

#### 3.2.3 Socio‐Demographic Factors

This study used the following socio‐demographic variables: *gender, age by birth year, school type/school level, mother's highest education, family structure, family's financial situation*, and *ethnic background*.

One's gender was asked according to the two official genders: *female or male*. The age of respondents was calculated by subtracting the year of response (2019) from the year of birth. The school level was assessed as “*Secondary school*” or “*Vocational school*”. The mother's highest educational lev was inquired by options: “Comprehensive school”, “Secondary high school or vocational school”, “Secondary high school or vocational school and vocational education”, and “University, University of Applied Sciences or other college education”. In this study, the responses were classified into three categories: *Comprehensive school, Secondary or vocational school, and Higher education at university or University of Applied Sciences*. We excluded the variable of father's education level due to a higher incidence of missing values. Additionally, prior research [18] indicates that mother's education level is a more significant predictor of children's developmental outcomes. *Family structure* was assessed by asking the respondents to identify the adults with whom they lived. Four categories were used: *With parents, Living in two homes with one parent after separation, With one parent, or Some other environment*. The students were asked if their family's financial situation was *Very good, Fairly good, Moderate, Fairly poor, or Very poor* to assess the financial situation. In the original survey, respondents' foreign background was measured by four category options: “Finnish”, “Other parent foreign”, “Foreign, born in Finland”, and “Foreign, born abroad”. In this study, we categorised the variable into three groups: *Finnish* (including *Other parent foreign*), *Foreign, born in Finland*, or *Foreign, born in another country*.

### 3.3 Confounding Variables

The confounding factors were *self‐perceived health* and *self‐perceived anxiety*. Previous research reveals that all these factors are usually associated with the health and well‐being among children and adolescents [19, 20, 21]. So, we wanted to control them when examining the associations between a young person's caring relationship and psychosocial symptoms.

*Perceived health* was assessed by a one‐item question: How is your general health? The response options were on a 5‐point scale: *Very good, Fairly good, Average, Fairly bad, or Very bad*. A 7‐item Generalised Anxiety Disorder Scale (GAD‐7) assessed *self‐rated anxiety*. This scale asks about the frequency of seven symptoms of GAD over the past 2 weeks; the response options were “Not at all”, “Several days”, “More than half of days,” or “Nearly every day”. This study categorised self‐perceived anxiety into two groups: *No anxiety/Slight anxiety* or *At least moderate anxiety*. The original GAD scale ranges from 0 to 21 points, with a score of fewer than 10 indicating “No anxiety”, and a score of at least or more than 10 but < 15 indicating “Slight anxiety”. Scores between 16 and 21 mean *Severe anxiety* [21]. This study compares respondents who scored at least 15 points on the Generalised Anxiety Disorder (GAD) measure with those who scored fewer than 15 points. GAD‐7 has been validated in several studies and in the adolescent population [22, 23].

### 3.4 Data Analysis

The statistical analyses were conducted using IMB SPSS Statistics 27 and R version 4.0.2. Frequencies and percentages were used to describe the sample. Cross‐tabulation and *χ* ^2^ tests were performed for categorical variables to establish the association between socio‐demographic variables and the caring relationship (Table 1) and the associations between caring relationships and psychosomatic symptoms (Table 2). Adjusted and unadjusted odds ratios were then calculated using logistic regression analyses to describe and test the associations between caring relationships and psychosomatic symptoms (Table 3). In the logistic regression analyses, we used two‐category variable of psychosomatic symptoms: (1) “Fewer than two symptoms per week” and (2) “At least two symptoms per week”. The logistic regression model was adjusted with socio‐demographic factors, that is, gender, age, the mother's education, family structure, the family's financial situation, and ethnic background, as well as confounding factors, that is, self‐perceived health and anxiety. *p* values for ORs were calculated using Fisher's exact test and AORs were calculated using logistic regression. Results from the logistic regression analyses are presented as odds ratios (ORs and AORs) and their 95% confidence intervals. The level of statistical significance was set at *p* < 0.001 due to the large number of respondents.

**TABLE 1:** Characteristics of the participants and their association with caring relationship.

Characteristics	Helping and caring a family member				
	None % (*n*)	Monthly or yearly % (*n*)	Weekly/daily (*n*)	Total % (*n*)	*p*
**All students**	86.0 (56,866)	8.9 (5475)	5.1 (3107)	100.00 (61,448)	
**Gender**					
Male	87.3 (25,128)	8.0 (2315)	4.7 (1357)	46.9 (28,800)	< 0.001
Female	85.0 (27,738)	9.7 (3160)	5.4 (1750)	52.1 (32,648)	
**Age**					
18	86.0 (24,817)	9.0 (2613)	5.1 (1460)	47.0 (28,890)	0.771
16–17	86.2 (28,105)	8.8 (2868)	5.1 (1652)	53.0 (32,625)	
**Current school level**					
Upper secondary school	86.9 (36,853)	8.7 (3689)	4.4 (1865)	68.9 (42,407)	< 0.001
Vocational school	84.1 (16,069)	9.4 (1792)	6.5 (1247)	31.1 (19,108)	
**Mother's highest education**					
Comprehesive	80.5 (1834)	11.3 (257)	8.3 (188)	3.8 (2279)	< 0.001
Upper secondary or vocational	85.7 (24,568)	9.0 (2567)	5.3 (1521)	47.5 (28,656)	
Higher education	87.0 (25,594)	8.7 (2549)	4.4 (1292)	48.8 (29,435)	
**Family structure**					
With parents	87.0 (35,333)	8.1 (3293)	4.9 (1981)	67.0 (40,607)	< 0.001
With parents (divorced)	86.3 (8201)	9.3 (880)	4.4 (420)	15.7 (9501)	
With one parent	84.0 (4212)	10.2 (512)	5.8 (293)	8.3 (5017)	
Other environment	80.6 (4455)	12.8 (707)	6.6 (365)	9.1 (5527)	
**Financial situation of the family**					
Very good	88.7 (12,229)	6.9 (955)	4.4 (603)	22.5 (13,787)	< 0.001
Fairly good	87.5 (24,474)	8.3 (2318)	4.2 (1174)	45.7 (27,966)	
Moderate	83.7 (12,826)	10.4 (1589)	5.7 (913)	25.0 (15,238)	
Fairly poor	77.4 (2727)	13.8 (487)	8.8 (308)	5.8 (3522)	
Very poor	70.1 (427)	15.3 (93)	14.6 (89)	1.0 (609)	
**Ethnical background**					
Finnish	86.4 (48,680)	8.8 (4951)	4.8 (2726)	95.9 (56,357)	< 0.001
Foreign, born in Finland	85.4 (923)	6.8 (73)	7.9 (85)	1.8 (1081)	
Foreign, born in other country	78.5 (1039)	12.1 (160)	9.4 (124)	2.3 (1323)	
**Self‐perceived health**					
Very good	88.7 (15,420)	7.1 (1233)	4.2 (729)	28.6 (17,382)	< 0.001
Fairly good	86.2 (26,202)	8.9 (2715)	4.9 (1492)	50.1 (30,409)	
Average	83.0 (9076)	11.0 (1199)	6.0 (655)	18.0 (10,930)	
Fairly or very bad	77.2 (1546)	13.2 (265)	9.6 (192)	3.3 (2003)	
**Anxiety**					
Not at all or slight anxiety	88.8 (24,936)	7.0 (1972)	4.2 (1186)	46.5 (28,094)	< 0.001
At least moderate	83.7 (27,062)	10.5 (3397)	5.8 (1861)	53.5 (32,320)	

*Note*: Pearson's Chi‐squared test *p* ≤ 0.001.

**TABLE 2:** Cross‐tabulation of psychosomatic symptoms and caring relationship.

Caring relationship					
Symptoms during past half year	None% (*n*)	Monthly or yearly % (*n*)	Daily or weekly % (*n*)	Total (*n*)	*p*
**Neck or shoulder pain**					
Seldom or never	38.7 (20,403)	31.2 (1701)	31.6 (979)	23,083	< 0.001
Once a month	31.9 (16,819)	32.8 (1791)	31.3 (972)	19,582	
Once a week	18.8 (9897)	22.8 (1246)	22.0 (681)	11,824	
Almost daily	10.6 (5588)	13.2 (720)	15.1 (469)	6777	
**Lower back pain**					
Seldom or never	50.1 (26,403)	42.50 (2316)	40.8 (1260)	29,979	< 0.001
Once a month	31.1 (16,360)	33.5 (1828)	31.9 (985)	19,173	
Once a week	12.6 (6611)	15.6 (851)	15.5 (480)	7942	
Almost daily	6.2 (3282)	8.4 (456)	11.8 (365)	4103	
**Stomachache**					
Seldom or never	50.1 (26,342)	41.8 (2274)	41.1 (1273)	29,889	< 0.001
Once a month	36.6 (19,212)	40.8 (2217)	39.4 (1219)	22,648	
Once a week	10.1 (5319)	12.6 (685)	13.4 (416)	6420	
Almost daily	3.2 (1674)	4.8 (263)	6.1 (189)	2126	
**Trouble falling asleep or waking up during the night**					
Seldom or never	44.5 (23,439)	35.9 (1953)	38.0 (1178)	26,570	< 0.001
Once a month	24.5 (12,885)	25.5 (1389)	22.6 (700)	14974	
Once a week	19.8 (10,399)	23.8 (1296)	21.1 (655)	12,350	
Almost daily	11.3 (5924)	14.8 (808)	18.3 (566)	7298	
**Headache**					
Seldom or never	36.5 (19,213)	29.3 (1597)	29.3 (909)	21,719	< 0.001
Once a month	35.1 (18,469)	35.5 (1936)	32.0 (991)	21,396	
Once a week	21.4 (11,268)	25.8 (1405)	25.7 (796)	13,469	
Almost daily	7.1 (3745)	9.5 (519)	13.0 (402)	4666	
**Tiredness or dizziness**					
Seldom or never	23.8 (12,532)	16.2 (882)	20.6 (356)	14,052	< 0.001
Once a month	29.4 (15,490)	28.6 (1557)	25.5 (787)	17,834	
Once a week	28.5 (14,992)	32.7 (1780)	27.3 (844)	17616	
Almost daily	18.3 (9653)	22.5 (1226)	26.6 (823)	11,702	

*Note*: Pearson's chi‐squared test.

**TABLE 3:** Unadjusted and adjusted logistic regression models: Factors associated with psychosomatic symptoms (ref. Fewer than two symptoms per week vs. At least two symptoms per week).

Factors	OR	95% CI	*p*	AOR	95% CI	*p*
**Caring**						
Not at all vs. at least monthly	1.390	1.294, 1.494	< 0.001	1.052	0.966, 1.145	0.197
Not at all vs. weekly	1.854	1.701, 2.022	< 0.001	1.455	1.309, 1.616	< 0.001
**Gender**						
Male vs. Female	3.850	3.658, 4.052	< 0.001	2.344	2.202, 2.496	< 0.001
**Age**						
18 vs. 16–17	0.895	0.857, 0.935	< 0.001	0.981	0.932, 1.033	0.474
**Current school**						
Upper secondary vs. vocational school	0.987	0.942, 1.035	0.600	1.344	1.263, 1.430	< 0.001
**Mother's highest education**						
Comprehensive school vs. upper secondary or vocational	0.674	0.607, 0,749	< 0.001	0.890	0.783, 1.013	0.076
Comprehensive school vs. Higher education	0.611	0.550, 0.678	< 0.001	0.964	0.846, 1.100	0.584
**Family structure**						
Living with both parents vs. Living with both parents after separation (two homes)	1.213	1.140, 1.290	< 0.001	1.079	1.004, 1.160	0.038
Living with both parents vs. Living with one parent	1.748	1.624, 1.880	< 0.001	1.154	1.057, 1.260	< 0.001
Living with both parents vs. Living with another environment	1.924	1.796, 2.061	< 0.001	1.200	1.099 1.309	< 0.001
**Financial situation of the family**						
Very good vs. Fairly good	1.312	1.238, 1.410	< 0.001	0.926	0.858, 0.999	0.046
Very good vs. Moderate	2.134	1.99, 2.283	< 0.001	1.057	0.973, 1.148	0.189
Very good vs. Fairly poor	3.720	3.397, 4.073	< 0.001	1.404	1.255, 1.570	< 0.001
Very good vs. Very poor	6.060	5.107, 7.191	< 0.001	1.855	1.493, 2.302	< 0.001
**Ethnic background**						
Finnish vs. Foreign, born in Finland	1.307	1.121, 1.525	< 0.001	1.130	0.938, 1.356	0.191
Finnish vs. Foreign, born in another country	1.386	1.209, 1.588	< 0.001	1.155	0.973, 1.366	0.095
**Self‐perceived health**						
Very good vs. Fairly good	3.288	3.036, 3.560	< 0.001	2.029	1.857, 2.220	< 0.001
Very good vs. Average	10.722	9.872, 11.645	< 0.001	4.875	4.436, 5.364	< 0.001
Very good vs. Fairly bad or very bad	30.277	27.006, 33.944	< 0.001	12.384	10.862, 14.129	< 0.001
**Self‐perceived anxiety**						
Nor at all or slightly anxiety vs. at least moderate anxiety	8.344	7.823, 8.899	< 0.001	4.242	3.936, 4.575	< 0.001

*Note:* Unadjusted and adjusted odd ratios in the logistic regression of psychosomatic symptoms on caring relationship factors (and their 95% confidence intervals). Pearson's chi‐squared test *p* ≤ 0.001.

## 4 Results

### 4.1 Characteristics of Respondents

Altogether, the study included 61,448 students. Of this group, 9% had experience of caring relationships at least monthly and 5% at least weekly (Table 1). Of these, 52% were females and 47% were males. The participants were nearly evenly split, with 53% aged 16–17 and 47% aged 18. Students were from secondary high schools (69%) or vocational schools (31%). Almost half (49%) of the respondents reported that their mother's education was higher at the university level; nearly half (48%) had upper secondary or vocational school studies; and 4% had comprehensive school. Two‐thirds (67%) of the respondents lived with both parents, 16% lived in two homes after their parents' separation, 8% lived with one parent, and 9% lived in some other environment. The family's financial situation was reportedly very or fairly good by 68% of students, moderate by 25%, and fairly or very poor by 7%. Most (96%) of the students had a Finnish background, 2% had a foreign background but were born in Finland, and 2% had a foreign background and were born in another country. Self‐perceived health was reported as very or fairly good by 79%, moderate by 18%, and fairly or very bad by 3%.

### 4.2 Socio‐Demographics and Caring Relationship

Table 1 describes the association between the socio‐demographic factors and the caring relationship. Females reported that they cared for their loved ones slightly more than males. The vocational students expressed that they participated in caring for a loved one more frequently than high schoolers.

The mother's lower education level was associated with the caring relationship. If the respondent's mother had a background in lower education, they experienced caring situations more monthly, weekly, or daily than those whose mother's education was at university or the University of Applied Sciences.

Family structure was also associated with a caregiving relationship. Young people who lived with one parent or in environments other than with their parents had caregiving responsibilities more often than those living with both parents or in two homes after a divorce.

Young people who reported more frequent involvement in caregiving activities also more often perceived their family's financial situation as fairly or very poor.

A foreign background was also associated with a caring relationship. Students born abroad participated more frequently in caring for a loved one than young people with a Finnish background.

### 4.3 Confounding Variables and Caring Relationship

Young carers reported their health condition to be poorer than those with no caring relationship. Self‐rated anxiety was also associated with a caring relationship, for example, 6% of respondents reporting at least moderate anxiety had a caring relationship at least weekly.

### 4.4 Associations of the Psychosomatic Symptoms and Caring Relationship

#### 4.4.1 Psychosomatic Symptoms and the Caring Relationship

Table 2 describes the associations between a caring relationship and single psychosomatic symptoms. Students who had a caring relationship reportedly suffered more often from all inquired psychosomatic symptoms than those who did not. For instance, the students in a daily or weekly caring relationship reported more frequently lower back pain (12%) than their peers without caring responsibilities (6.0%) and stomachache (6%) than their peers without caring responsibilities (3%). Similarly, their caregivers were almost twice as likely to report almost daily headaches (13%) than the non‐caregivers (7%). In addition, daily or weekly carers had neck and shoulder pain (15%), sleeping problems (18%), and tiredness or dizziness (27%) approximately a third more often than students without caring responsibilities.

#### 4.4.2 Psychosomatic Symptoms, Caring Relationship, and Socio‐Demographic Factors

Being in a caring relationship more frequently was associated with a higher frequency of all single psychosomatic symptoms (*p* < 0.001). The distribution of daily symptoms was almost double among students in a caring relationship than those without.

The associations of a caregiving relationship and background factors with psychosomatic symptoms as neck or shoulder pain, lower back pain, stomachache, sleeping problems, headache and tiredness were further examined using logistic regression analysis. A binary variable was used for psychosomatic symptoms: (1) Fewer than two symptoms per week and (2) At least two symptoms per week.

In the unadjusted model (Table 3), the caregiving relationship, several socio‐demographic factors, and both confounding variables were statistically significantly associated with self‐perceived psychosomatic symptoms. A daily or weekly caregiving relationship had higher odds (OR = 1.85, 95% CI = 1.70, 2.02) of psychosomatic symptoms than no caregiving relationship. Lower age (OR = 0.90, 95% CI = 0.86, 0.94) showed lower odds for psychosomatic symptoms. Female gender (OR 3.85, 95% CI = 3.66, 4.05); living in a non‐intact family, such as in two homes after divorce (OR 1.24, 95% CI = 1.14, 1.29); or with only one parent (OR 1.75, 95% CI = 1.62, 1.88); very poor financial situation (OR 6.06, 95% CI = 5.11, 7.19); and student being born in another country (OR 1.39, 95% CI = 1.21, 1.59) showed higher odds for psychosomatic symptoms. Also, the confounding factors, that is, fairly or very bad self‐perceived health (OR 30.28, 95% CI = 27.01, 33.94) and at least moderate anxiety (8.34, 95% CI = 7.82, 8.90), showed higher odds for psychosomatic symptoms.

In the adjusted model (Table 3), when the 10 variables, that is, caring relationship, gender, age, current school, mother's educational level, family structure, family's economic situation, ethnic background, self‐perceived health, and self‐perceived anxiety were entered into the same model, the associations remained almost as similar as in unadjusted analyses, with some exceptions. Compared to those without, *those with a weekly or daily caring relationship (AOR 1.46, 95% CI = 1.31, 1.62) had greater odds of psychosomatic symptoms when all socio‐demographic and confounding factors were controlled simultaneously. Furthermore, in the adjusted model, female gender (AOR 2.34, 95% CI = 2.20, 2.50), living with only one parent (AOR 1.15, 95% CI = 1.06, 1.26), living in another environment (AOR 1.20, 95% CI = 1.10, 1.31), fairly poor (AOR 1.40, 95% CI = 1.26, 1.57) or very poor family economic situation (AOR 1.87, 95% CI = 1.50, 2.32), fairly or very bad self‐perceived health (AOR 12.38, 95% CI = 10.86, 14.13), and at least moderate self‐perceived anxiety (AOR 4.24, 95% CI = 3.94, 4.58) were associated with higher odds for psychosomatic symptoms*. In the adjusted model, students' age and ethnic background, family structure living in two homes as well as mothers' highest education were not associated with psychosomatic symptoms.

## 5 Discussion

This study, with a large nationally representative population‐based sample, showed that psychosomatic symptoms are more common among 16–18‐year‐old students in a caregiving relationship than without it. Several socio‐demographic variables tested independently, that is, gender, age, mother's education, family structure, family's economic situation, and ethnic background, were associated with psychosomatic symptoms. Furthermore, higher levels of self‐perceived health and anxiety were both associated with increased caregiving responsibilities among young people. After controlling for all the socio‐demographic and confounding factors, this study identified that a caring relationship was still associated with a higher frequency of perceived psychosomatic symptoms.

Young carers are more likely to have psychosomatic symptoms than their non‐carer counterparts. These findings support previous studies [2, 24], showing that young carers find their care work physically demanding and emotionally draining, which certainly indicates the strain of the caring relationship. Our study also showed that caregiving responsibilities were associated with poorer self‐perceived health. The findings align with Becker and Becker's [24] study, where young carers identified adverse physical and mental health outcomes directly related to caring. Previous research shows that experiencing frequent non‐specific health complaints like psychosomatic symptoms indicates low well‐being [25].

Our study showed that the family's poor financial situation seemingly relates to the caregiving relationship. One explanation may be that the factors causing caring situations may also cause employment problems, thus affecting the family's financial situation. Similarly, according to Becker [1], young adult carers experience significant financial hardships due to caring for and living in a low‐income family where there is physical or mental ill health, disability, and alcohol or drug misuse. If the family income is very low, there is strong evidence of poverty and social exclusion for all family members and young carers [24].

Our study indicated that self‐perceived anxiety was associated with caring responsibilities. An interesting finding was that 10.5% of those with at least moderate anxiety had caring responsibilities at least monthly, and 5.8% of this group had caring responsibilities at least weekly. By comparison, the figures were 7% and 4%, respectively, for those with no or slight anxiety.

Our results support those of Wepf and Leu [4] who found that young carers experienced lower levels of well‐being and higher levels of perceived stress than their peers. In their further analysis, young carers' well‐being was not abnormal compared to all young people, but being a young carer was associated with higher levels of perceived stress. Indicators of family instability also predicted mental health outcomes independently of being a young carer [4]. Gallagher et al.'s [9] study also showed that young carers living in Europe have higher depressive symptoms than young people in Europe who are not carers. In practice, for example, in school health care, these symptoms and possible caregiving roles should automatically be asked during health examinations, and support should be provided. Experience shows that young carers mostly do not recognise their own caring role. Identifying young carers is a crucial first step, and a common definition of young carers is needed, as well as greater opportunities for young adults to identify themselves as a carer [26]. Therefore, the following question should be asked indirectly: Do you support/help a family member or close one with an illness, accident, frailty, or something else? [27].

Some additional intriguing findings concerning socio‐demographic factors' association with psychosomatic symptoms showed that females experienced psychosomatic symptoms more often than their male counterparts, aligning with the results of previous studies where girls have reported health problems [28] more often and have lower self‐perceived health [29] than males. Moreover, females may be more sensitive to noticing things and expressing their symptoms. To better understand how caregiving affects young people's well‐being, future research should explore whether factors such as gender moderate this relationship by testing interaction effects.

We also found that living in family arrangements other than with both parents may increase the prevalence of psychosomatic symptoms among students. Previous research has shown that family structure impacts children's well‐being. The divorce situation of parents alone can be stressful for children [30]. Changes in the family structure may also increase parents' psychological workload and affect their ability to parent, leading to changes in other important areas of life, such as where parents live and work, friendships and neighbourhood relationships, children's schools, and care homes [31, 32].

To our knowledge, this is the first population‐based study to examine associations between socio‐demographic factors and psychosomatic symptoms among young carers. Our results suggest that a young person's caring role increases psychosomatic symptoms, although the impact of several background and confounding factors is controlled.

This new evidence highlights the importance of considering the caring relationship and the other socio‐demographics mentioned when assessing and discussing the well‐being of students. Therefore, school stakeholders, such as school health nurses and teachers, need to (a) recognise the students who are in a caring relationship and (b) offer them suitable support to live a decent life and fulfil their developmental tasks. In general, including all family members, especially children, in health care would be wise when considering the coping of the whole family.

Further research is warranted to focus on developing ways for schools to identify, recognise, and support young carers. Developing and assessing the tools and methods for schools to recognise and support young carers would be useful. In Finland, the Alisa Project—developed in collaboration with healthcare professionals—has introduced the Young Carers' Concern Cards, a practical tool designed to help identify and map the roles and needs of young carers [33].

There is also a need to identify what kind of support young carers need, where and at what time they need it, whether they are getting it and if not, why the support is not given. Further research is also needed to explore what a caring role means in daily life for these young people and the challenges it poses for their lives and futures. This study showed that a caring role at least weekly for a significant one is seemingly more common among students born in another country (9%) than among students with Finnish backgrounds (5%). It would be valuable to know more about this cultural perspective and how students with a foreign background perceive the caring relationship and its consequences for them.

Psychosomatic symptoms experienced during youth may have far‐reaching effects on later life according to previous research [34]. Future research should consider the potential long‐term effects of psychosomatic symptoms experienced in young people, including their possible associations with academic underachievement, reduced occupational success, and increased risk of future health problems.

## 6 Limitations Including Reliability and Validity

Due to the study's cross‐sectoral design, establishing a cause‐and‐effect relationship is not possible. A longitudinal study would allow monitoring of the development of the situation over time. Due to the large amount of data, there may also be statistically significant but clinically insignificant results. Therefore, the level of statistical significance was set at *p* < 0.001.

One of the strengths of this study in terms of *content validity* is that the caregiving‐related question was developed in collaboration with international young carer researchers. This ensured alignment with definitions established in previous research. However, some limitations should be acknowledged. Firstly, the potential caregiving relationship was assessed using a single item, meaning the more detailed aspects of the caregiving role were not explored. Secondly, young people may not always identify their actions as caregiving, particularly when supporting someone close to them.

The large population‐based sample supports the *external validity* of the study by enhancing the generalizability of the findings. This was the first time the caregiving background of young people has been investigated with a nationally representative sample in Finland. However, some cautions should be considered. Young carers may be underrepresented in this study, as those who did not respond or who dropped out of the survey could include a higher proportion of students with caregiving responsibilities. Furthermore, young carers may be overrepresented among students who were absent during data collection [35].

Several factors support the *reliability* of the study. Firstly, the School Health Promotion Study has been conducted since the 1990s, consistently reporting similar results. This demonstrates the stability of the measurement over time [36]. However, the stability of the question regarding the caregiving role should be further assessed in future data collections. Secondly, the anonymous and voluntary nature of the study encourages honest responses, thereby enhancing the reliability of the data [37].

## 7 Conclusion

This study provides—for the first time—unique population‐based evidence of the associations between socio‐demographic factors, caring relationships, and psychosomatic symptoms in vocational and upper secondary school settings. The main study result is that taking over caring tasks at least weekly increased the odds of having psychosomatic symptoms. The association remained, although several socio‐demographic and confounding factors were controlled. It is important for education and health professionals to recognise the existence of young carers and to be aware of their significant caregiving roles.

## Author Contributions

Ella Eronen‐Levonen, Katja Joronen, and Riitta Suhonen designed the study. Miko Pasanen and Ella Eronen‐Levonen conducted data analysis, with Miko Pasanen also serving as the statistical expert. Ella Eronen‐Levonen drafted the manuscript. Critical revisions for important intellectual content were made by Ella Eronen‐Levonen, Miko Pasanen, Kaisa Mishina, Riitta Suhonen, Agnes Leu, and Katja Joronen. Katja Joronen and Riitta Suhonen supervised the project.

## Ethics Statement

The ethical approval of the original study has been granted. Participants were informed about the study, the voluntary nature of participation, and the ability to withdraw from the study or deny answering any question without reason per local regulations. Respondents anonymously completed a classroom‐administered questionnaire under their teacher's supervision, most likely adding to the study's response rate. The proportion of missing responses was low. Data confidentiality was ensured. Good scientific practice was followed throughout the research. The whole questionnaire is on the THL website (https://thl.fi/en/web/thlfi‐en/research‐and‐development/research‐and‐projects/school‐health‐promotion‐study/questionnaires). The multi‐professional panels carefully reflect and evaluate all the scales in the School Health Survey. H. Kivimäki (personal communication, 2023). The researcher applied for permission to use the original research material to the extent necessary. Permission was obtained from the Finnish Social Science Data Archive. The researcher has used and stored the data carefully, and there is no information available that would endanger the data protection of the informants.

## Conflicts of Interest

The authors declare no conflicts of interest.

## Data Availability

The data that support the findings of this study are available on request from the corresponding author. The data are not publicly available due to privacy or ethical restrictions.
